# *Leishmania* lipophosphoglycan: how to establish structure-activity relationships for this highly complex and multifunctional glycoconjugate?

**DOI:** 10.3389/fcimb.2014.00193

**Published:** 2015-01-21

**Authors:** Claire-Lise Forestier, Qi Gao, Geert-Jan Boons

**Affiliations:** ^1^INSERM U1095, Faculté de Médecine, University of Aix-MarseilleMarseille, France; ^2^Complex Carbohydrate Research Center, Department of Chemistry, University of GeorgiaAthens, GA, USA

**Keywords:** *Leishmania* glycoconjugates, lipophosphoglycan, LPG structure, LPG function, chemical synthesis, LPG structure-activity relationships

## Abstract

A key feature of many pathogenic microorganisms is the presence of a dense glycocalyx at their surface, composed of lipid-anchored glycoproteins and non-protein-bound polysaccharides. These surface glycolipids are important virulence factors for bacterial, fungal and protozoan pathogens. The highly complex glycoconjugate lipophosphoglycan (LPG) is one of the dominant surface macromolecules of the promastigote stage of all *Leishmania* parasitic species. LPG plays critical pleiotropic roles in parasite survival and infectivity in both the sandfly vector and the mammalian host. Here, we review the composition of the *Leishmania* glycocalyx, the chemical structure of LPG and what is currently known about its effects in the mammalian host, specifically. We will then discuss the current approaches employed to elucidate LPG functions. Finally, we will provide a viewpoint on future directions that this area of investigation could take to unravel in detail the biological activity of the specific molecular elements composing the structurally complex LPG.

## The *Leishmania* surface coat

Like all the parasites of the Trypanosomatid family, *Leishmania* is characterized by the presence of a glycocalyx covering the entire parasite surface (Ferguson, 1999). The surface coats of these different trypanosomatid parasites exhibit a significant diversity in composition. However, all of the surface-bound molecules of this family share a common structural feature, which is that they all contain a highly conserved glycosylphosphatidylinositol (GPI)-anchor motif. Notably, this type of GPI-lipid anchor is unusual and structurally very different from those found in mammalian cells (Mcconville and Ferguson, 1993).

Unlike other trypanosomatids in which the glycocalyx is primarily composed of GPI-anchored glycoproteins, the glycocalyx of the *Leishmania* promastigote stage is dominated by GPI-anchored phosphoglycosylated glycans. Lipophosphoglycan (LPG) represents one of the most abundant promastigote-specific surface glycoconjugates, with approximately 5 × 10^6^ copies/cell (Turco and Descoteaux, 1992). The glycosylinositol phospholipids (GIPLs) also termed free GPI, constitute a complex family of abundant low-molecular-weight molecules, with approximately 10^7^ copies/cell. Three different types of GIPL molecules have been described based on the nature of their glycan moiety (Mcconville et al., 1993). In the GIPL of type 1, the glycan part is structurally similar to that of the LPG glycan core, whereas in the GIPL of type 2, the glycan part is related to that of the GPI-anchored glycoprotein. The GIPLs of type 3 exhibit features of type 1 and 2. The membrane-bound proteophosphoglycans (mPPGs) represent a distinct family of GPI-anchored protein-linked glycans that express a phosphoglycan domain structurally similar to LPG. The mPPGs are significantly expressed at the promastigote parasite surface but to a lesser proportion than LPG and GIPLs (Ilg, 2000). Last, one of the major GPI-anchored glycosylated proteins present at the promastigote plasma membrane is GP63, with around 5 × 10^5^ copies/cell. Importantly, the composition of the *Leishmania* surface glycocalyx changes dynamically during the life cycle of the parasite. When infective, promastigote parasites differentiate into obligate intracellular amastigotes in the infected mammalian host cell, the expression of LPG is drastically downregulated. In contrast, GIPLs and PPGs remain highly expressed throughout the parasite life cycle (Turco and Sacks, 1991). Notably, the PPGs continue to be produced in amastigotes, but as free macromolecules rather than membrane-associated ones (Bahr et al., 1993).

The glycoconjugates of the *Leishmania* promastigote membrane are evenly distributed over the entire parasite surface. They form a highly hydrophilic barrier easily detected as an electron-dense material using electron microscopy. Its thickness can reach up to 15 nm due to the length of the LPG polysaccharide chain and potentially up to several hundred nanometers due to the lengths of the mPPGs (Ilg, 2000). Because of their abundance, structural uniqueness and specific distributions, the *Leishmania* membrane glycoconjugates are believed to play important functions in the mammalian host. Among these compounds, LPG has attracted considerable attention because its clear implication in multiple activities that favor parasite virulence.

## *Leishmania* LPG structure

**Figure 1:**
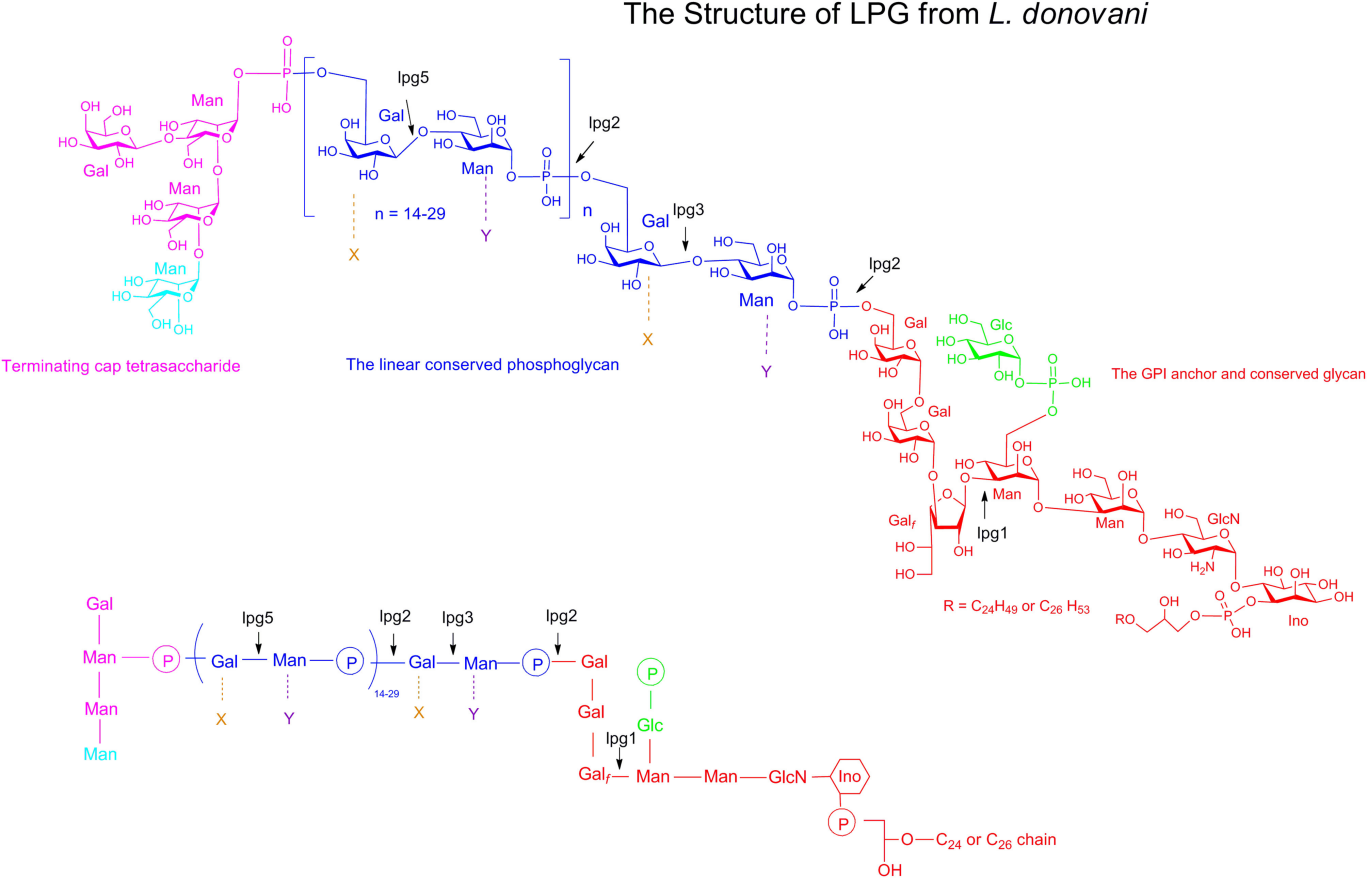
**Structure of the *Leishmania* LPG**. **Top** and **bottom panels** show two different representations of the LPG structure of *Leishmania* parasite. LPG is constituted of four key domains. The GPI anchor and the glycan core are shown in red. The linear conserved phosphoglycan chain is in blue. The terminating oligosaccharide cap is shown in pink. In each domains, the residues that are not conserved among the LPG of the different Leishmania species are represented in a different color (Man, X, Y and Glc). The Glucose phosphate branched on the mannose residue of the glycan core (in green) is present in the LPG of *L. donovani, L. mexicana* and some subspecies of *L. major* but absent in some other subspecies. The linear phosphoglycan chain of *L. donovani* can be substituted with different residues. X represents the substitutent group express in the LPG *L. mexicana, L. major* and *L. tropica*. Y is a substitutent present in the LPG of *L. aethopica*. Gal, galactose; Man, Mannose; Glc, glucose; Galf, galactofuranose; GlcN, glucosamine; Ino, inositol.

*Leishmania* LPG, is a highly complex macromolecule composed of four distinct domains: a GPI anchor, a glycan core, a linear phosphoglycan chain (PG) and a terminating oligosaccharide cap (Figure 1) (Turco and Descoteaux, 1992). The GPI anchor domain consists of an alkyl phosphatidylinositol having a single saturated C_24−26_ aliphatic chain (Ferguson, 1999). The LPG glycan core is a heptasaccharide comprising two galactopyranosides, a galactofuranoside (Gal_f_), two mannosides and a glucosamine residue attached to inositol. The glycan core is linked to a linear PG that consists of 15–40 phosphodisaccharide (Galβ1,4Manα1-PO4) units. Finally, LPG is terminated by a di-, tri- or tetrasaccharide consisting of galactose and mannose assembled as Manα1,2Manα1 or as Galβ1,4(Manα1,2)Manα1 depending on the *Leishmania* species.

The lipid anchor, glycan core and the linear PG moieties that constitute the LPG are identical in all *Leishmania* species (Descoteaux and Turco, 1999). Despite conservation of these domains, however, LPG exhibits substantial heterogeneity, with important parasite stage- and species-modifications found in the oligosaccharide cap and in the substituents groups branched on the linear PG (Turco et al., 2001; De Assis et al., 2012). Stage-specific variations are observed throughout the parasite life cycle such that LPG undergoes considerable structural modifications in the PG and the terminating cap during parasite metacyclogenesis (Sacks et al., 1990, 1995). In the PG domain, the number of repeating units increases such that the metacyclic promastigote LPG is significantly longer than the procyclic promastigote LPG. In the oligosaccharide cap, change is made with the replacement of the galactoside residue by an arabinopyranoside residue. Species-specific variations occurring in the PG domain of the LPG are one of the main features of this virulent factor. Three types of LPG have been described depending on the nature of the side chain residues and on the site of substitution occupied by these residues in the PG domain. The LPG of *L. donovani* has no side substitution in the PG and therefore remains linear (Sacks et al., 1995). The LPGs of *L. major, L. mexicana, L. infantum*, and *L. tropica* are glycosylated at the C3 position of the galactose in the linear PG (Soares et al., 2002) and the LPGs of *L. aethiopica* are frequently mannosylated at position C2 of the mannose. Additionally, the variability in the sugar residues that branch on the PG domain increases significantly the level of LPG complexity. Finally, intraspecific LPG variability has been observed among similar *Leishmania* species obtained from different field isolates (Coelho-Finamore et al., 2011). Such stage-specific polymorphisms and intra- and interspecies variations have been involved in the survival of *Leishmania* inside the sand fly, more precisely in the selectivity, permissivity, and competence of a given sand fly vector for particular *Leishmania* strains (Dobson et al., 2006, 2010; Volf et al., 2014). However, the role and the biological relevance of LPG polymorphism in the mammalian host has been poorly understood.

Given the structural complexity and heterogeneity of LPG molecules, the identification of the molecular elements responsible for its biological activity have only partially been resolved.

## Role of LPG in *Leishmania* interactions with the immune system

The LPG-enriched glycocalyx of *Leishmania* constitutes the primary interface of the host-parasite interactions that take place in the dermis of the mammalian host immediately after parasite inoculation by the sandfly vector. Consequently, LPG is the first target for immune detection and at the same time a barrier protecting the parasite from the attack of the host immune system.

LPG has been shown to circumvent the lysis of the parasite by the host complement system. It acts either by sterically preventing the attachment of complement molecules or by directly inactivating the assembly of a functional complement complex at the promastigote surface (Puentes et al., 1989, 1990).

LPG has been shown to favor intracellular parasite survival by interfering with the pro-inflammatory host cell responses via binding of Toll-like receptor (TLR) 2 and 4 on macrophages and NK cells (Becker et al., 2003; De Veer et al., 2003; Kavoosi et al., 2009; Rojas-Bernabe et al., 2014). LPG-TLR interactions induce ERK phosphorylation while suppressing p38 MAP kinase phosphorylation, modulate the production of reactive oxygen species and nitric oxide and inhibit pro-inflammatory cytokine secretion (Chandra and Naik, 2008; De Assis et al., 2012). These studies showed that the integrity of the lipid anchor as well as the length of the PG domain of LPG are involved in the magnitude of LPG-mediated host cell activation via TLR2, as procyclic promastigotes are weaker stimulators than metacyclic promastigotes. The level of LPG expression is also been considered as a critical parameter of this specific host cell stimulation pathway (Srivastava et al., 2013). Given that the GPI anchor of *trypanozoma cruzi* has been implicated in TLR2-mediated activation, it is conceivable than the analogous site in *Leishmania* LPG play a similar function (Campos et al., 2001). Finally, by comparing the LPG from *L. braziliensis* and *L. infantum* a recent study reveals that interspecies LPG structural polymorphism has a significant impact on host cell stimulation via TLR (De Assis et al., 2012; Ibraim et al., 2013) Despite these advances, it remains to be determined which LPG motifs are exactly implicated in TLR binding and further immune cell stimulation.

**Figure 2:**
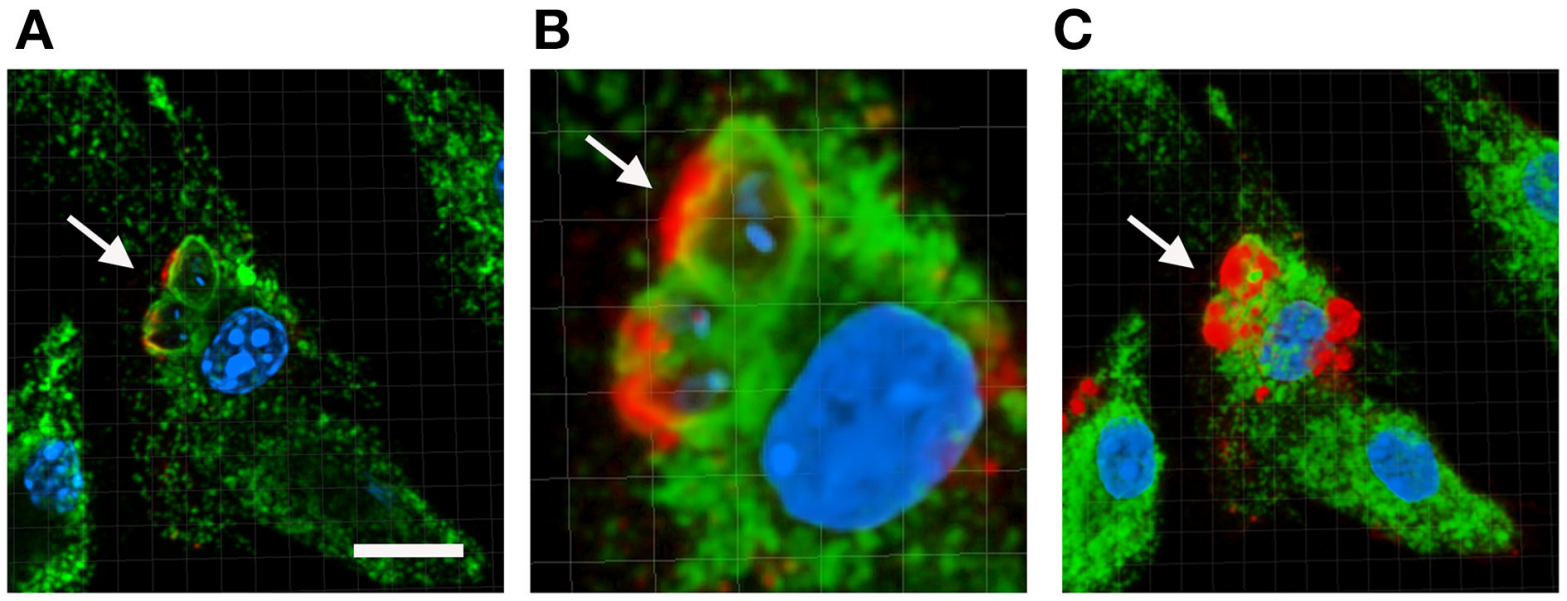
**Localization of LPG at the membrane of the *Leishmania*-containing phagosome**. Bone marrow macrophages were infected with *L.donovani* promastigote for 1 h at 37°C then macrophages were washed to remove extracellular parasites and incubated in new medium for 24 h. Infected cells were processed for immunofluorescence staining. The LPG molecules were stained using the anti-PG antibody CA7AE (Red), the lysosome and phagosome compartments were stained using the anti-LAMP-1 antibody (Green), the host cell and the parasite nuclei were stained using the Hoechst dye (Blue). Images are analyzed using the Imaris software. **(A)** Represents a single Z section of 0.7 μm. **(B)** Represents a zoomed image area of **(A)**. Panel **(C)** is a 3D reconstruction of the entire Z stack. Arrow points to two Leishmania-containing vacuoles showing LPG at the phagosomal membranes. Scale bar, 10 μm.

The role of LPG during parasite internalization and multiplication within the host cell has been extensively studied but they have yielded contradictory results. For instance LPG has been shown to delay the maturation of the parasite-containing phagosome by preventing its fusion with lysosomes while some groups have demonstrated that such phenomenon does not occur (Desjardins and Descoteaux, 1997; Forestier et al., 2011). In parallel, LPG has been found to block the assembly of NADPH oxidase and prevent recruitment of proton ATPases at the phagosomal membrane (Lodge and Descoteaux, 2005; Vinet et al., 2009). This function has been attributed to the localization of LPG at the membrane of the *Leishmania*-containing phagosome (Dermine et al., 2005; Winberg et al., 2009) (Figure 2). Therefore investigating a potential correlation between the intracellular localization of LPG inside the host cells and a particular biological function would require to be explored. Since the first observation of the presence of the PG disaccharide repeat units of the LPG at the surface of infected host cells (Tolson et al., 1990), a phenomenon that was later confirmed by our group (Forestier, 2013), the fate and pattern of trafficking of LPG during the infection process remain elusive. Given this lack of knowledge, monitoring LPG trafficking in the host cell and in the host organism during the infection process will be key to better understand LPG functions in its mammalian host. Furthermore, investigating whether chemical modifications of LPG occur during the infection process will help unraveling the importance of the distinct LPG structural motifs on its biological functions.

## LPG as a promising vaccine candidate

Despite its lack of strong immunogenicity but because of its unique structure, dense distribution and accessibility, *Leishmania* LPG has been considered as an attractive vaccine target (Goel et al., 1999). Early vaccine studies indicated that purified LPG provides protection in mice against challenge with virulent parasites. These studies have demonstrated that LPG-mediated protection can be obtained with LPG alone and that its efficacy depended on the integrity of the LPG molecule and could be modulated by the use of adjuvants (Handman and Mitchell, 1985; Russell and Alexander, 1988; Mcconville and Ferguson, 1993; Karanja et al., 2011). Contradictory results have arisen in more recent studies, in both protective and disease-promoting effects associated with LPG vaccination have been observed. These studies have revealed the importance of the immunization route in the vaccine outcome, with subcutaneous LPG injection failing to protect mice against *L. amazonensis* but intranasal administration of LPG showing to be protective (Pinheiro et al., 2005, 2007). Although the mechanisms of LPG-mediated immunization are yet not known, LPG has been shown to activate T cells and favor a Th1 immune response thus mediating protection against the intracellular stage of *Leishmania* (Handman and Mitchell, 1985; Moll et al., 1989; Moll and Rollinghoff, 1991; Tonui et al., 2003; Amprey et al., 2004). Paradoxically, a recent study showed that LPG vaccination, depending on the dose of LPG, induces the expression of the inhibitory receptors PD-1 and PD-L2 on T cells and macrophages respectively, therefore preventing proper protection against leishmaniasis (Martinez Salazar et al., 2014). Given the structural complexity of LPG, it remains unknown which feature of this glycoconjugate, independently or as a part of the whole macromolecule, is involved in the effective immunization process. Therefore, it is important to identify the functionally relevant molecular elements of LPG with the goal of developing artificial and well-designed LPG-based vaccines (Routier et al., 1999; Hewitt and Seeberger, 2001; Astronomo and Burton, 2010; Topuzogullari et al., 2013).

## Approaches to assess LPG functions

The involvement of *Leishmania* LPG in virulence has been confirmed in non-physiological conditions, using purified LPG molecules tested on macrophages *in vitro* and, in a more biologically relevant context, using parasites defective in specific steps of the LPG biosynthesis pathway.

Although the use of purified LPG has been very valuable to unravel LPG functions, it also has several drawbacks. Among these is the complicated purification procedures that it requires and along with it the difficulty of obtaining pure LPG preparations devoid of various contaminants. More problematic is the possibility that LPG preparation may become contaminated with trace amount of endotoxin, a problem commonly faced with the purification of molecules and that will greatly bias the host cell immune response. Finally, the use of purified LPG does not reflect the physiological conditions experienced in host cell-parasite interaction, and the dose of LPG used in such artificial functional assays may not replicate that encountered in actual physiological conditions.

Genetic approaches rely on the identification of genes encoding for enzymes that are involved in LPG biosynthesis, on the disruption of these target genes in *Leishmania* and on the analysis of the phenotypes and functions of such null mutants (Beverley and Turco, 1998). This type of studies has led to the generation of parasites displaying LPG molecules that are truncated at different levels of their polysaccharide moieties. *Leishmania* parasites were generated with mutations in the LPG1, LPG2, LPG5, and LPG3 genes that, respectively, encodes a galactofuranosyltransferase involved in the synthesis of the LPG glycan core specifically (Ryan et al., 1993), a Golgi GDP-mannose transporter required for the synthesis of the PG domain common to LPG and mPPG (Ma et al., 1997), a Golgi UDP-Gal transporter critical for the synthesis of the PG (Capul et al., 2007a,b) and the *Leishmania* homolog of a mammalian endoplasmic reticulum chaperone required for complete PG synthesis (Descoteaux et al., 2002). As a result, lpg1^−^ parasites express intact GIPLs and mPPG molecules, whereas LPG molecules have a truncated glycan core and no PG domain neither terminal oligosaccharide cap (Spath et al., 2000). In contrast, lpg2^−^ and lpg5^−^ parasites express normal GIPLs but these parasites lack all PG domains including those of LPG and mPPG; however LPG molecules have a normal glycan core (Spath et al., 2003; Liu et al., 2009). Finally, lpg3^−^ parasites express LPG molecules truncated after the first mannoside residue of the first disaccharide unit composing the PG domain (Descoteaux et al., 2002).

These null-mutant parasites and others recent LPG-mutants (Phillips and Turco, 2014) provide powerful tools for identifying the functions of the LPG in a context that closely mimics the natural course of infection, including a physiological concentration of LPG interacting with the host. Importantly, analyses of these mutants have been proven to discriminate efficiently between the roles of LPG and those of other related glycoconjugates, including mPPG, that express similar polysaccharide domains. Nevertheless, the nature of the lpg1, lpg2, lpg3, and lpg5 mutants offers the possibility of assessing the biological activity of only the LPG polysaccharide domains including the last three sugar residues of the glycan core, the phosphoglycan disaccharide repeating units and the oligosaccharide cap. To the best of our knowledge, the functional impact of the species-specific substituents of the PG domain and the nature and structure of the GPI anchor has not been yet investigated. Most likely, this reflects the difficulty inherent in engineering parasites deficient in such specific and essential structural motifs. Indeed, such an approach requires first the identification of the specific genes involved in the addition of the substituent to the linear PG and involved in GPI-anchor biosynthesis and then the mutation of these genes, to obtain viable parasites expressing modified glycoconjugates. Previous attempts to generate GPI-null *Leishmania* have demonstrated that this domain is critical for parasite viability and infectivity (Ilgoutz et al., 1999; Garami et al., 2001). In contrast, parasites deficient exclusively in the assembly of the GPI-anchored glycoproteins but not in the expression of LPG or GIPLs retain their capacity to grow and remain virulent (Mensa-Wilmot et al., 1994; Hilley et al., 2000; Zufferey et al., 2003). However, these studies were not able to discriminate between the GPI-anchor of LPG and other related structures carried by the other glycoconjugates featuring the *Leishmania* surface. To achieve a complete map of LPG structure-function relationships, it is critically required to identify genes involved specifically in the different steps of LPG biosynthesis, so that new LPG mutants may be generated.

Nevertheless, despite the valuable information gained by these studies, the main limitation of such genetic approaches is the limited opportunity they afford to investigate in detail and independently the relative roles of the different structural motifs of highly complex LPG molecules (oligosaccharide cap, phosphopolysaccharide chain, glycan core, GPI anchor and fatty acid chain). Although LPGs display high levels of heterogeneity, whether the molecular composition of the LPG motifs plays a distinct role in LPG function has never been determined. Therefore, there is an urgent need to explore the relative implications and contributions of the different LPG molecular elements to its biological activity.

## Future approaches

In our point of view, one of the priorities for future research into the functions of LPG is to elucidate the structure-activity relationship of this membrane-bound glycoconjugate. The ultimate goal is to identify which LPG motifs are associated with specific effects in the mammalian host. Such informations are expected to bring key new insights into the mechanism of action of this macromolecule.

To overcome some aspects of the limitations linked to the genetic approaches, alternative methods that will aim to dissect the LPG functional groups at the molecular level will need to be developed. Chemical synthesis of such structurally complex glycoconjugates may be one of the promising experimental approaches for investigating the distinct functions of each of the structural elements that compose the multifunctional LPG molecules. With technological advances in chemistry, new synthetic strategies and methods for the chemical synthesis of highly complex glycoconjugates are currently developed which will make conceivably the synthesis of LPG feasible (Astronomo and Burton, 2010). These chemical synthesis methods will allow the design and production of panels of synthetic LPG variants having independent molecular variations within its four distinct domains. Such synthetic LPG compounds will be crucial tools for investigating the functional relevance of the molecular elements of LPG. Using chemical synthesis one could expect to address and elucidate the importance of (i) the composition of the fatty acid chain of the GPI-anchor; (ii) the glycan core; and (iii) the repetitive units in the PG domain. Comparisons among the chemically well-defined collection of LPG variants will allow us to assign a molecular motif to a biological function. Such chemical synthesis methodology-based research could be extended to the study of all the others GPI-anchored glyconconjugates expressed at the cell surface of *Leishmania* and others related parasites. This will include GIPLs that unlike LPG, is expressed both at the promastigote and amastigote parasite stages and for which information about its role in the mammalian host remains very limited.

This type of approach will significantly advance our knowledge of the structurally complex LPG. Significantly, it will help to dissect the various LPG domains and attribute a precise function to each specific LPG elements, thereby establishing a causal structure-activity relationship. Ultimately, such chemistry-based strategy will open new venue to the development of LPG-based therapeutics agents against Leishmaniasis using synthetic and biologically relevant molecular elements of LPG.

### Conflict of interest statement

The authors declare that the research was conducted in the absence of any commercial or financial relationships that could be construed as a potential conflict of interest.
